# Neuroform EZ Stenting for Symptomatic Intracranial Artery Stenosis: 30 Days Outcomes in a High-Volume Stroke Center

**DOI:** 10.3389/fneur.2019.00428

**Published:** 2019-05-16

**Authors:** Haowen Xu, Tao Quan, Osama O. Zaidat, Dapu Chen, Zibo Wang, Yongjie Yuan, Baojun Yan, Hong Lu, Sheng Guan

**Affiliations:** ^1^Departments of Interventional Neuroradiology, First Affiliated Hospital of Zhengzhou University, Zhengzhou, China; ^2^Departments of Endovascular Neurosurgery and Stroke, St. Vincent Mercy Medical Center, Toledo, OH, United States; ^3^Department of Neurosurgery, Second People's Hospital of Pingdingshan, Pingdingshan, China; ^4^Departments of Neurology, First Affiliated Hospital of Zhengzhou University, Zhengzhou, China

**Keywords:** atherosclerosis, complication, endovascular procedure, intracranial stenosis, stent

## Abstract

**Objective:** To test whether Neuroform EZ stent placement combined with the modified techniques in symptomatic severe intracranial stenosis (ICAS) would result in lower rates of peri-procedural complications of intracranial stenting.

**Methods:** We retrospectively reviewed the clinical data from 71 consecutive patients who underwent Neuroform EZ stent placement combined with the modified techniques for symptomatic severe ICAS at our institute between January 2016 and October 2017. The primary outcomes were ipsi-lateral ischemic stroke, intra-cerebral hemorrhage, or death within 30 days after stenting. The secondary outcome was technical success.

**Results:** The technical success rate was 100%. The mean pre and post-stent stenoses were 84.2% ± 9.1% (median 85%, IQR75% to 90%) and 16.9% ± 10.2 % (median 15%, IQR 10% to 25%). The frequency of ipsi-lateral stroke, intra-cerebral hemorrhage, or death within 30 days was 0%.

**Conclusions:** The combined use of Neuroform EZ stent placement and the modified techniques for symptomatic severe ICAS is technically feasible and safe, with very low peri-procedural complications. Further studies are required to assess the long-term results of this approach.

## Introduction

The Stenting and Aggressive Medical Management for Preventing Recurrent Stroke in Intracranial Stenosis (SAMMPRIS) trial demonstrated the superiority of aggressive medical therapy over percutaneous transluminal angioplasty and stenting (PTAS), with a 30-day rate of stroke or death of 5.8% in the medical management group compared with 14.7% in the Wingspan stenting group (*P* = 0.02). (1) The Vitesse Intracranial Stent Study for Ischemic Stroke Therapy (VISSIT) trial also revealed a higher rate of peri-procedural complications among patients treated with balloon-expandable stent than that of patients treated with medical therapy (24.1 vs. 9.4%, *P* = 0.05) (2). However, for patients with severe ICAS and poor collateral circulation, PTAS remains an alternative under the premise of a fairly low incidence of peri-procedural complications. In recent years, there have been remarkable advances in both endovascular technique and stent choice. Vajda et al. reported that 8.5% (16/189) of patients who were treated with undersized balloon angioplasty combined with Enterprise stent placement experienced stroke or death within 30 days after procedure (3). This result was similar to another retrospective study by Feng et al. (4), who used a similar technique with the same stent to treat symptomatic ICAS. Of the 44 patients treated with Enterprise stent placement, three (6.8%) ischemic and 1 (2.2%) hemorrhagic strokes occurred during the peri-procedural period.

Since 2014, we have modified the interventional skills and patient selection of PTAS, resulting in a low rate of peri-procedural complications with high technical success rate. The self-expanding, open-cell Neuroform EZ stent (Stryker Neurovascular, Freemont, CA) may be better suited to the PTAS than the Wingspan stent, partly because the former is a microcatheter-delivered stent, allowing easier access to the target vessels and stent deployment (5). Some experts thought access is an important prognostic factor for successful intracranial stenting (6).

The aim of this study was to investigate whether Neuroform EZ stenting using the modified techniques would be able to result in lower rates of peri-procedural complications, compared to the prior published data using the Wingspan stent with regular endovascular technique.

## Methods

Written informed consent was obtained from all participants. In addition, our local institutional ethics review board (the First Affiliated Hospital of Zhengzhou University) approved the research protocol for this retrospective study.

We conducted a retrospective review from prospectively collected databases of consecutive patients with symptomatic ICAS of the major intracranial arteries who were treated with Neuroform EZ stenting using the modified techniques in our institution. From January 2016 through October 2017, a total of 71 patients with 72 symptomatic ICAS were treated with Neuroform EZ stent placement combined with the modified techniques and met the following inclusion criteria: (1) severe (70–99%) ICAS involving the intracranial ICA, the M1 segment of MCA (M1), intra-dural VA, and BA; (2) recurrent transient ischemic attack (TIA) or non-disabling stroke (modified Rankin Scale score ≤ 3) despite antiplatelet therapy; (3) at least one of the following cardiovascular risk factors, including current smoking, hypertension, type 2 diabetes mellitus, and dyslipidemia; (4) a duration of ≥ 1 week from the latest ischemic symptom onset; (5) age ≥35 years. Patients with any of the following were excluded: (1) non-atherosclerotic stenosis (such as dissection, vasculitis, and moyamoya disease); (2) history of cerebral hemorrhage; (3) brain contrast-enhanced T1-weighted magnetic resonance imaging (MRI) showed apparent parenchymal contrast enhancement within the infarcted area; (4) contraindication to endovascular procedures (renal failure, coagulopathy, and contrast allergy).

### Procedural Technique

**Figure 1:**
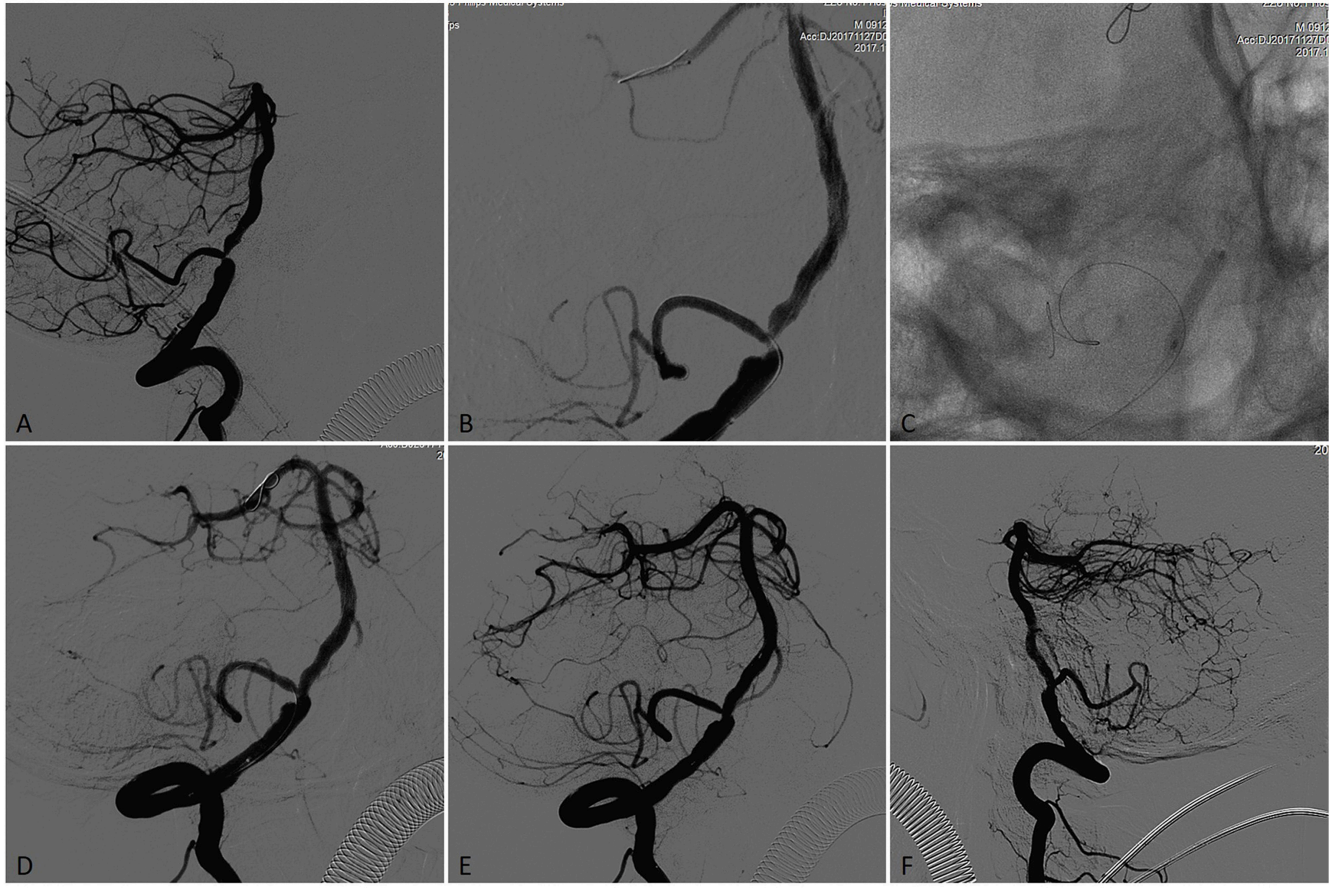
A patient presented initially with recurrent posterior circulation territory TIAs on anti-platelet therapy, including diplopia. **(A)** Subtracted angiogram in the working angle demonstrated left vertebral artery occlusion and a concentric severe (75%) stenosis in the V4 segment of the right vertebral artery with the posterior inferior cerebellar artery (PICA) origin from the stenotic lesion. **(B)** A 0.008 inch microwire was placed in PICA aimed to protect it during the PTAS and a 0.014 inch microwire placed in P2 segment of right posterior cerebral artery (PCA) with the microwire tip manipulated a U- shaped loop inside the vessel. **(C)** The stenotic lesion was dilated with a 3.0 × 15-mm Gateway balloon. **(D)** Vertebral angiography showed significant improvement of the stenosis immediately after angioplasty. **(E)** Microcatheter angiography showed no vessel perforation, dissection and thrombus occurred in the distal artery where the microwire tip placed. **(F)** A 4.0 × 15-mm Neuroform EZ stent was deployed across the target lesion, Final angiography showed near 15% residual stenosis.

A dose of 300 mg of aspirin and 75 mg of clopidogrel was given daily for all patients at least 3 days prior to the procedure. All procedures were performed under general anesthesia via a 6F or 8F Guide Catheter. Intraprocedure unfractionated heparin was administered at ~70 units/kg as an IV bolus to achieve an activated clotting time of 250 to 300 s. If the vascular structure was quite tortuous, distal intracranial catheters (DICs), such as the Navien (Medtronic Neurovascular, Irvine, CA) and DAC (Distal Access Catheter; Stryker Neurovascular, Freemont, CA) were used. Under a road-map, a 0.014 inch Traxcess microwire (Microvention, Tustin, CA) with exchange length was placed distal to the stenosis. The tip of microwire should be placed in the distal straight vessel branching point and small side branches (ascertained by microcatheter angiography), and the wire tip would be manipulated into a U-shaped loop inside the vessel (Figure 1). A Gateway balloon catheter (Stryker Neurovascular) was advanced over the microwire across the target lesion. The balloon diameter was sized to 80% (undersized to 60%-80% for “high risk” plaque) of the normal vessel size. The criteria of “high risk” plaque included: (1) atherosclerotic plaques distribute in superior and (or) dorsal wall of M1 or dorsal and (or) lateral wall of BA; (2) important branch originating from the plaque; (3) age ≥80 years. Angioplasty was performed with a slow (at least 2 min), gradual inflation of the balloon to a pressure between 6 and 12 atm. As for angulated lesions, the proximal and distal segment of a stenotic lesion should be dilated with balloon inflation, respectively. In the case of a straight lesion, a long balloon angioplasty (15 mm, in general) was recommended to decrease the risk of the balloon migration during the inflation. Following angioplasty, the balloon was removed and an Excelsior XT-27 microcatheter (Stryker Neurovascular) was placed through the lesion over the existing exchange-wire and a microcatheter angiography was then performed to detect if vessel perforation occurred in the distal vessel where the microwire tip was placed. A Neuroform EZ stent was delivered through the Excelsior XT-27. Deployment of the stent was accomplished by withdrawal of the microcatheter while fixing the stabilizer. An additional balloon angioplasty would have been performed if ≥50% residual stenosis was observed. Technical success was defined as completion of Gateway balloon angioplasty and stent placement across the target lesion with final residual stenosis <50%. Before discharge, all patients underwent clinical examination and were instructed to take 100 mg aspirin daily for life and 75 mg clopidogrel daily for 3 months.

### Data Collection and Follow-Up

The observations indices included age, sex, symptoms and vascular risk factors of the patients, location of the target lesion, normal vessel diameter adjacent to the lesion, stenotic degree, stenotic length, residual stenosis after procedure, as well as occurrence, type, and severity of peri-procedural complications. All patients received a face-to-face or telephone interview by trained personnel to evaluate the clinical adverse events at 30 days after stenting. If necessary, brain MRI or computer tomography (CT) was obtained in patients with new symptoms.

The primary outcomes were occurrence of ipsi-lateral ischemic stroke, intra-cerebral hemorrhage, or death within 30 days after procedure. The secondary outcome was procedural success, which was defined as the completion of balloon angioplasty and stent placement across the target lesion with <50% immediate residual stenosis. Ischemic stroke was defined as a new focal neurologic deficit of sudden onset, lasting ≥24 h, which is not associated with a hemorrhage on CT or MRI of the brain. Hemorrhagic stroke was defined as a new brain hemorrhage, involving parenchymal hemorrhage, subarachnoid hemorrhage, or intra-ventricular hemorrhage that is associated with a seizure or with symptoms or signs lasting ≥24 h. Asymptomatic infarction was considered to be an adverse event but was not included as a primary end point.

### Statistical Analysis

The statistical methods used were predominantly descriptive. Continuous variables were expressed as mean (±SD) or as median with an interquartile range (IQR) for those with skewed distributions, while nominal variables are presented as percentages. For selected percentages, 95% confidence intervals were calculated by using the Clopper-Pearson (exact) method. The frequency of ipsi-lateral ischemic stroke, intra-cerebral hemorrhage, or death within 30 days was estimated using the Kaplan-Meier (product limit) method with pointwise CIs calculated using the log cumulative hazard transformation.

## Results

### Demographic Features, Qualifying Events

The study cohort included 71 patients (54 men and 17 women) with a mean age of 58.9 ± 8.2 years (range, 40–77 years). The indication for stenting was stroke in 52 cases (73.2%), TIA in 19 cases (26.8%). Two patients who developed ischemic stroke were treated with PTAS 33 and 42 days after their late-presenting stoke, respectively. The other 69 patients underwent Neuroform EZ stenting within 30 days after the qualifying event (range 7–29 days). Among the 72 stented intracranial arteries, 26 (36.1%) were located in the anterior circulation (M1 17 and ICA in 9) and 46 (63.9 %) were located in the posterior circulation (VA in 19 and BA in 22). The mean length of the treated stenotic lesions was 7.6 ± 2.2 mm (median 7 mm, quartiles 6 mm and 11 mm).

### Technical and Angiographic Results

**Figure 2:**
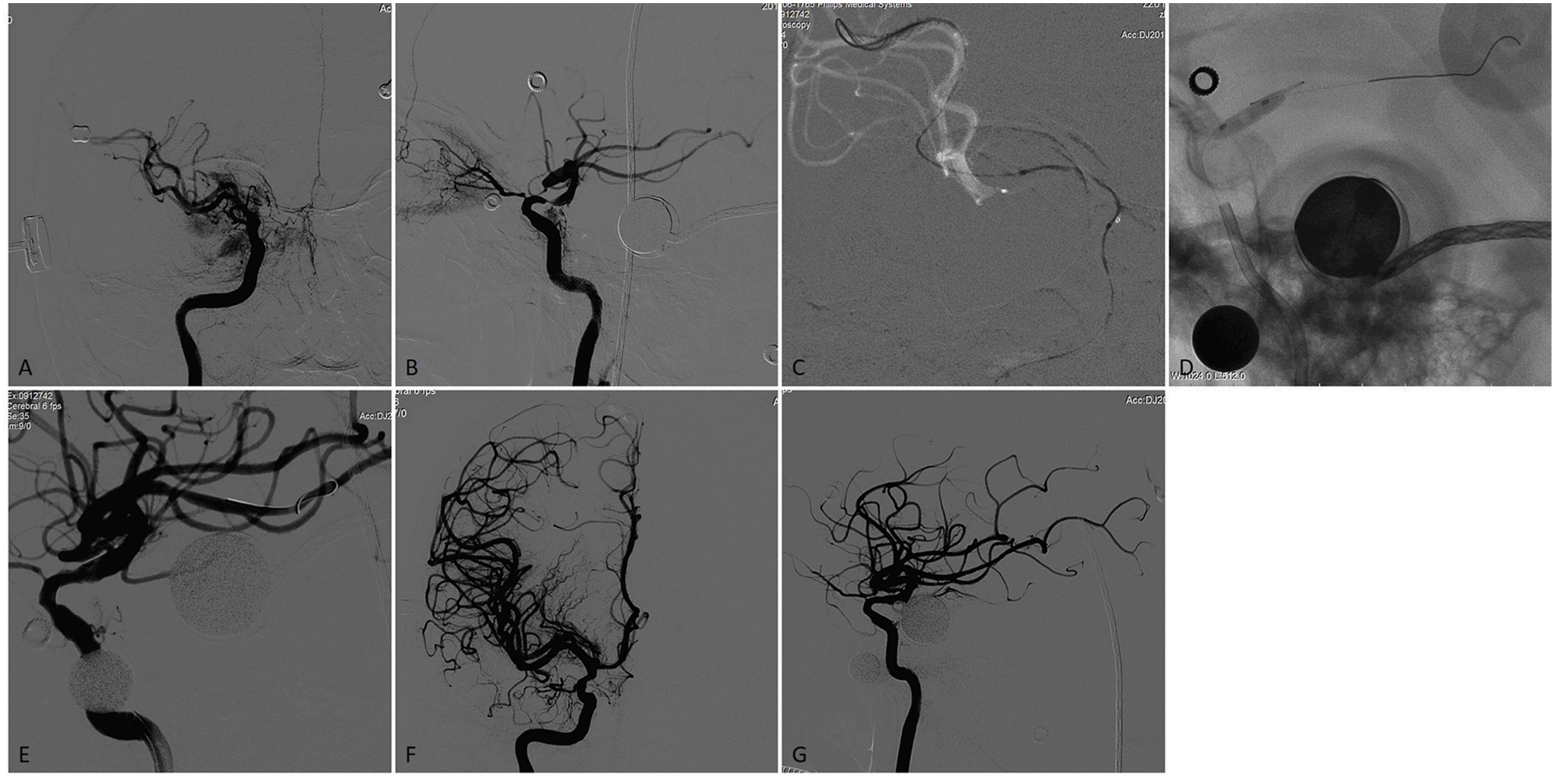
A patient who had a previous history stroke and presented again with recurrent TIAs recently. **(A,B)** Right internal carotid arteriogram on anteroposterior and lateral view demonstrated a focal severe (90%) stenosis of the right supraclinoid ICA with flow reduction in the distal territory. **(C)** A 0.014 inch microwire was anchored in the distal branch of the MCA with the microwire tip manipulated a U- shaped loop inside the vessel. **(D)** The stenotic lesion was dilated with a 3.5 × 9-mm Gateway balloon. **(E)** The lesion showed significant improvement of the stenosis immediately after angioplasty. **(F)** and G. A 4.5 × 15-mm Neuroform EZ stent was deployed across the lesion, Final angiography showed near 20% residual stenosis with flow augmentation in the distal territory.

The overall technical success rate was 100%. 9 patients required additional post-dilation and none of the patients received 2 or more stents placement. The mean pre-procedure stenosis was 84.2 ± 9.1% (median 85%, quartiles 75 and 90%) and the immediate mean post-stenting residual stenosis was 16.9 ± 10.2 % (median 15%, quartiles 1 and 25%). Arterial dissections at the target lesions occurred after balloon angioplasty in 6 cases, but all these dissections resolved after stent placement and did not result in any new neurologic deficits. Stent thrombosis during the peri-procedural period occurred in one case, which was successfully treated with intra-arterial administration of urokinase. Two patients developed symptoms of ischemia in the distribution of the treated vessels immediately after procedure, with contralateral hemiparesis. Both symptoms resolved completely within 1 hour after procedure. Vasospasm or dissections of the vessels where the U-shaped tip of microwire was placed were not encountered (Figure 2).

The frequency of ipsilateral ischemic stroke, intracerebral hemorrhage, or death within 30 days after stenting was 0%. Three patients developed a groin hematoma after the procedure and recovered after hemostasis by compression. The comparison of the clinical and imaging feature, 30-day outcomes between the SAMMPRIS trial and the present study is given in Table 1.

**Table 1:** The comparison of clinical and imaging feature, 30-day outcomes between the SAMMPRIS trial and the present study.

	**The SAMMPRIS trial**	**The present study**
No. of patients	224	71
Average age	61.0 ± 10.7	58.9 ± 8.2
Male/female	56.7%/43.3%	76.1/23.9%
No. of treated lesions	224	71
Severe stenosis (70–99%)	100%	100%
**LESION LOCATION**		
ICA	20.1%	12.7%
MCA (M1 segment)	41.1%	23.9%
VA	17.0%	26.8%
BA	21.9%	36.6%
**QUALIFYING EVENT**		
TIA	36.6%	26.8%
Stroke	63.4%	73.2%
Mean length of stenosis (mm)	6.7	7.6
**MEAN STENOSIS DEGREE**		
Pretreatment	79.7%	84.2%
Posttreatment	27.0%	16.9%
Technical success	92.0%	100%
**COMPLICATIONS**		
Ischemic stroke within 30 d	23 (10.2%)	0%
Hemorrhagic stroke within 30 d	10 (4.5%)	0%
Death within 30 d	5 (2.2%)	0%
Overall stroke or death within 30 d	33 (14.7%)	0%

## Discussion

The failure of SAMMPRIS and VISSIT trials was due primarily to high peri-procedural complications rather than a lack of flow restoration. Possible explanations for the disappointing results in both studies could be attributed to poor patient selection, limited experience, and device issues (7, 8). Therefore, how to effectively reduce complications is of a major concern in the current endovascular treatment of ICAS. In 2016, Gao et al. reported the short-term results of a large multicenter registry with 100 patients who underwent Wingspan stenting with some modifications in patient selection and procedural aspects. The technical success rate was 100% with a peri-procedural complication rate of 2% (9). It should be noted that the modifications between their study and our current study are dissimilar. In our study, a high technical success rate (100%) and very low rate of peri-procedural complication (0%) was achieved, which was lower than that of the Wingspan stenting arm and the medical treatment arm of the SAMMPRIS trial. This current study highlights our unique technical aspects of patient selection, endovascular procedure, and stent choice.

### Patient Selection

Since 2014, we have modified the patient selection of intracranial stenting. All patients who suffered from ischemic stroke due to ICAS and prepared for PTAS must not only meet the inclusion criteria of the SAMMPRIS trial, but must also not have an apparent parenchymal contrast enhancement within the infarcted area on contrast-enhanced T1-weighted MRI.

In March 2012, the Food and Drug Administration announced that intracranial stenting with the Wingspan stent system can be an option for patients with recurrent stroke despite medical therapy who have not had any new stroke symptoms 7 days before procedure (10). We think that the absolute 7-day intervals may not be necessarily accurate, because the injured tissue caused by ischemic stroke may not recover within such a short period. A study by Nahab et al. found that intracranial stenting soon after stroke was highly associated with a high incidence of post-procedure hemorrhage (11). They suggested that intracranial stenting should not be performed until the brain injuries get a remarkable recovery from an ischemic stroke.

Blood-brain-barrier (BBB) degeneration after cerebral ischemia may lead to hemorrhagic transformation (HT) (12, 13). The risk of HT was found to be associated with increased BBB permeability, and the BBB permeability within the infarct region is greatest at 6–48 h after the onset of acute ischemic stroke (AIS), and then decreased slowly (12). Strbian et al. found the BBB is continuously open for several weeks following cerebral ischemia (14). A study by Vo et al. demonstrated that parenchymal enhancement in acute stroke lesions, which is indicative of increased BBB permeability, was associated with a higher risk of HT (15).

The leading cause of hemorrhagic complications of intracranial stenting for ICAS is reperfusion hemorrhage, which could result in fatal consequences. In the SAMMPRIS trial, the incidence of reperfusion hemorrhage (including the unknown cause of post-procedure intra-parenchymal hemorrhage) was 3.1% (16). A recent published multicenter prospective trial of 100 patients treated with Wingspan stenting at 10 hospitals in China reported that the 1-month hemorrhagic complication rate was 0%. That trial recruited patients who had experienced an index ischemic event at least 3 weeks before intervention, which is longer than the recommended 7-day interval (range, 7–19 days) in the SAMMPRIS trial (9). However, it is still possible for the high BBB permeability following cerebral ischemia to not recover even after 3 weeks. In this current study, all 71 patients did not experience symptomatic HT within 30 days after the procedure, which may be partly attributed to our patient selection method. We infer that preoperative brain contrast-enhanced MRI, rather than a fixed time window, could more precisely prevent reperfusion hemorrhage after intracranial stenting.

### The Modified Endovascular Techniques and Procedure Safety

In a retrospective study, Vajda and his colleagues used the modified Bose method combined with Enterprise stent to treat 189 patients with ICAS (3). The so-called modified Bose method meant undersized balloon angioplasty followed by a slightly oversized self-expanding stent deployment. The size of the balloon was selected to reach about 80% of the diameter of the normal vessel. The procedural success rate was 100%, and the post-procedural complication rate within 30 days was 8.5%, which was lower than the rate of periprocedural complication reported in the SAMMPRIS trial (14.7%) and within the range of other Wingspan registries (6.1–20.6%) (17–21).

In the INTRASTENT and SAMMPRIS study, the risk of wire perforation was relatively high when treating intracranial ICA or MCA lesions, probably due to the fact that intracranial tortuous parent vessels were often seen in anterior circulation, especially in internal carotid artery siphon segment. The microwire would be apt to move back and forth while advancing the stent or microcatheter over the microware across the tortuous vessel, increasing the risk of artery injury (22). In the present study, we recommend having the tip of microguidewire placed in the vessel segment without rich small branches, thereby enabling the guidewire tip to be manipulated into a U-shaped loop inside the distal vessel. The aim of these maneuvers is to decrease the risk of distal microwire perforation. Before the advance of stent into the microcatheter, a microcatheter angiography was performed; if perforation occurred, the damaged vessel could be occluded in a timely with nbutyl cyanoacrylate (NBCA) to avoid serious complication.

The balloon is inflated across the stenotic lesion as a long balloon is more stable during inflation and easier to keep positioned, decreasing the incidence of balloon migration. The migration of a balloon can aggravate the “snow plow” effect, which is the main mechanism of perforator stroke after intracranial stenting (23). Therefore, as for the lesion in a relatively straight vessel, a long balloon (15 mm, in general) is recommended. However, the angioplasty that was performed by a long balloon across the lesion is not suitable for the angulated lesion. The long balloon may straighten the vessel during inflation, increasing the risk of a “snow plow” effect, dissection and vessel rupture. Under these circumstances, the proximal and distal segment of the lesion should be dilated with the proximal and distal end of the balloon, respectively. With respect to the “high risk” plaque, the angioplasty that is performed with a balloon undersized to ~60–80% of the normal vessel diameter may avoid vessel injury and minimize the risk of squeezing a plaque more distally into the perforator branches. All these measures may have contributed to the zero rate of perforator stroke in our trial, which was lower than the rate of the perforator stroke reported in the SAMMPRIS trial (6.7%, 15/224) and the study by Feng et al. (6.8%, 3/44) (4, 16).

### The Neuroform EZ Stent

Another aspect that could improve the safety and efficacy of intracranial stenting is the improvement in device design. The Wingspan stent is navigated across the lesion over an exchange-length microwire and may be not suitable for a target lesion with very tortuous anatomy. The en bloc delivering and deployment of the Wingspan stent in tortuous vascular pathways would be difficult, which may reduce the procedural success rate and increase the risk of wire perforation. The Neuroform EZ stent is an open-cell self-expandable nitinol stent, which is delivered through a 0.027-inch microcatheter system. Furthermore, this microcatheter delivery system could provide good navigability and accessibility in the tortuous vessel. In addition, the bumpers allow the Neuroform EZ to be moved backward and forward prior to unsheathing to achieve optimal positioning and microcatheter stability prior to deployment (5). Deployment of a Neuroform EZ stent is possible in almost every tortuous segment of major intracranial arteries. These features of the Neuroform EZ system seem to translate into lower complication rates. Some studies have demonstrated a higher technical success rate with a microcatheter delivered stent [Enterprise stent (94–100%) (3, 4, 24) Solitaire stent (100%) (25) than with Wingspan stent (91.2–100%) (17–23)].

The Neuroform EZ stent also provides other advantage in comparison with the Wingspan stent. The latter has twice the radial force than the former. One of the potential mechanisms of in-stent restenosis (ISR) is stimulation of intimal hyperplasia by the radial force of the stent. Compared with Wingspan, the neointimal hyperplasia occurs less in Enterprise stent placement which provides low radial force. Feng et al. reported a lower rate of ISR using Enterprise stent (6.8% restenosis during a mean 22 months of follow-up) (4). The ISR rate of PTAS with Wingspan was reported to range between 7.5 and 31.2% (26). The outward radial force of Neuroform EZ is slightly higher than that of Enterprise. Theoretically, the ISR rate may be lower in the Neuroform EZ stent placement compared to the Wingspan stenting.

There were some limitations in this study. Firstly, although the primary outcome rate is lower than in other comparable trials, the outcome definition and ascertainment methodology are not uniform between trials. Secondly, our study does not provide any information regarding the mid- or long-term results in connection with both clinical events and the ISR rate. After all, these results are critical evaluating indicators of the therapeutic efficacy of intracranial stenting. Thirdly, it is a one-institute based, non-randomized, retrospective study, which likely introduces selection bias. The low complication rates from a retrospective study might not necessarily be true in a prospective trial. Therefore, a multicenter, prospective cohort trial is still needed to certify our results.

## Conclusions

This retrospective study demonstrated that a very low periprocedurl complication rate and a high technical success rate of intracranial stenting for symptomatic ICAS could be achieved by using Neuroform EZ stent placement combined with the modified techniques. The long-term outcome and ISR rate remain unknown.

## Ethics Statement

This study was carried out in accordance with the recommendations of committee of first affiliated hospital of zhengzhou university with written informed consent from all subjects. All subjects gave written informed consent in accordance with the Declaration of Helsinki. The protocol was approved by the committee of first affiliated hospital of zhengzhou university.

## Author Contributions

HX and SG were responsible for designing the study and revising the manuscript. TQ, OZ, and HL were responsible for analyzing the data and drafting the manuscript. YY, ZW, and DC were responsible for data collection. HX, TQ, and BY were responsible for the study supervision and coordination.

### Conflict of Interest Statement

The authors declare that the research was conducted in the absence of any commercial or financial relationships that could be construed as a potential conflict of interest.

## Abbreviations

ICAS: intracranial atherosclerotic stenosis
SAMMPRIS: Stenting and Aggressive Medical Management for Preventing Recurrent Stroke in Intracranial Stenosis
VISSIT: Vitesse Intracranial Stent Study for Ischemic Therapy
ICA: internal carotid artery
MCA: middle cerebral artery
BA: basilar artery
VA: vertebral artery
DSA: digital subtraction angiography.
